# Hypolipidemic and antioxidative effects of curcumin on blood parameters, humoral immunity, and jejunum histology in *Hy-line* hens

**Published:** 2013

**Authors:** Javad Arshami, Mohammad Pilevar, Mohammad Aami Azghadi, Ahmad Reza Raji

**Affiliations:** 1 *Department of Animal Sciences, College of Agriculture, Ferdowsi University of Mashhad, Mashhad, I. R. Iran*; 2 *Department of Pathology, College of Veterinary Medicine, Ferdowsi University of Mashhad, Mashhad, I. R. Iran*

**Keywords:** Curcumin, Humoral Immunity, Hypolipidemic Agent, Jejunum Histology

## Abstract

**Objective:** *Turmeric* (*Curcuma Longa Linn*) is a medicinal plant that contains curcumin. There is a growing interest in using curcumin powder (CP) as feed additives for antioxidative and antimicrobial properties to improve human health. This study was conducted to determine the appropriate levels of CP on blood parameters, immunity, and histology of jejunum in hens.

**Materials and Methods:** A total of 200, 58-wk-old Hy-line hens were randomly distributed into 4 treatments (0%, 0.5%, 1.5%, and 2.5% CP or 0, 5, 15, and 25 g/kg feed, respectively) with 5 replicates (10 birds each) for 8 weeks using the completely randomized design. Blood samples were taken from 2 birds per replicate at weeks 61 and 65 to evaluate blood parameters. On weeks 63 and 65, two birds from each replicate received 0.5 ml SRBC (25%) injection in breast muscle and 7 days later, blood samples were collected to evaluate total Ig, IgG, and IgM titers in serum. Two hens were sacrificed at week 65 for the histological study of jejunum.

**Results:** Curcumin reduced triglycerides at 1.5% and 2.5% and cholesterol and LDL at 2.5% (p<0.05). Improvement of total Ig and IgG titers after the 1^st ^and 2^nd^ injections were observed. Muscle thickness in jejunum increased (p<0.05) at 1.5% curcumin and the length and surface area of villus were enhanced as well.

**Conclusion:** Our results demonstrate that curcumin can be used as an antioxidant at 1.5% and antilipidemic agent at 2.5% in diet.

## Introduction

Turmeric (*Curcuma Longa Linn*.) is a medicinal plant that contains curcumin, demethoxycurcumin, bisdemethoxycurcumin (Wuthi-Udomler et al., 2000 ▶), and tetrahydrocurcuminoids (Osawa et al., 1995 ▶), which are the active ingredients. Beneficial effects of curcumin in human and animal nutrition may include stimulation of body metabolism (Srinivasan, 2005 ▶), improvement of endogenous digestive enzyme secretion (Platel and Srinivasan, 2000 ▶), activation of immune response (Antony et al., 1999 ▶), antibacterial and antiviral (Singh et al., 2010 ▶), antioxidant (Toda et al., 1985 ▶), anti-Alzheimer’s disease (Park and Kim, 2002 ▶), anticancer (Kuttan et al., 1985 ▶), and anthelminthic actions (Gomez et al., 2002 ▶). The curcumin extract was found to have immunomodulatory and antioxidative activities in mice (Antony et al., 1999 ▶; Iqbal et al., 2003 ▶). Moreover, curcumin had greater impacts on preventing free radical damage compared with vitamin C, vitamin E, and superoxide dismutase (Toda et al., 1985 ▶). 

A recent study provided the first immunological evidence that dietary supplementation of curcumin enhances local innate immunity and induces higher protective immunity against *E. tenella *infection (Lee et al., 2009 ▶). A recent study showed that curcumin has protective effects against AFB1-induced toxicity by modulating red and white blood cell counts and Hb percentage to some extent (Sharma et al., 2011 ▶; Gowda et al., 2008 ▶). Animals fed with a high dose of curcumin (3 g/kg body weight) did not show any significant adverse effects (Shankar et al., 1980 ▶). It has been stated that 1.5 g curcumin per person per day can be used as a pain relief or wound healing medicin (Sharma et al., 2005 ▶). The potent anti-inflammatory and anti-arthritis effects of curcumin volatile oil on joint damage has been compared with cortisone and phenylbutazone (Chandra and Gupta, 1972 ▶). However, these medicines are associated with toxicity in bone and blood, whereas curcumin has no side effects. 

An earlier study showed that 0.5 g curcumin prominently enhanced the activity of intestinal lipase (80%), amylase (96%), and trypsin (154%) which play an important role in digestion and absorption of food (Platel and Srinivasan, 2000 ▶; Srinvasan, 2005 ▶). At present, *curcumin* is being used in human and animal diets to promote immunity and health status. However, as up to now, the most effective level has not been determined. Therefore, in this study, we used higher levels of curcumin to specify the hypolipidemic and antioxidative effects on blood parameters, humoral immunity, and jejunum histology in hens. 

## Materials and methods

**Animals and treatments**

This experiment was conducted at poultry research farm according to the rules and guidelines approved by the Animal Care and Use Committee of College of Agriculture, Ferdowsi University of Mashhad. A total of 200 Hy-line, W-36 laying hens were allotted to 4 treatments with 5 replicates (50 birds/treatment, 10 birds/replicate) using the completely randomized design. The *Curcumin* powder (CP) was purchased from local market (ZAMEN Co. INC, Mashhad, Iran). The birds recieved a basal diet (control) or a basal diet containing 5, 15, and 25 g curcumin powder/kg feed (0.5%, 1.5%, and 2.5%, respectively) for 8 weeks (58-65 wk old). The birds were housed in cages under similar condition receiving *ad libitum* feed and water. 

**Blood parameters**

Blood samples (1 mL/bird) were taken from the brachial vein into heparinzed tubes from 40 randomly selected birds (2 birds/replicate, 10 birds/treatment) at 61 and 65 weeks of age. Plasma was obtained from blood samples by centrifugation (10 min at 3000 rpm) and stored at -20 ºC. The frozen plasma was allowed to thaw at 22 ºC prior to analysis. Plasma total protein, albumin, creatine kinase, triglyceride, cholesterol, LDH, glucose, phosphorus, and calcium were determined by autoanalyzer (Technicon RA1000, Bayer Diagnostics, Puteaux, France) using available commercial kits (Pars Azmun Co. INC, Tehran, Iran). 

**Humoral immune respons**

The SRBC (sheep red blood cell) test was performed in order to assay the specific antibody (Ab) titer. To evaluate the Ab responses, 2 birds/treatment were randomly selected and inoculated with SRBC (25% suspension in PBS, 0.5 mL/bird in both left and right breast muscle) at the end of weeks 63 and 65. One week after 1^st ^and 2^nd^ inoculations (weeks 64 and 66), 0.5 mL blood samples were collected from the main brachial vein using a 2 mL syringe with a 22-gauge needle and were placed into heparinized tubes to evaluate the primary and secondary Ab responses. The sera were inactivated by heat at 56 °C for 30 min. The total and mercaptoethanol-resistant (IgG) Ab titers against SRBC were determined by agglutination (Cheema et al., 2003 ▶). 

The serum (50 µL) was added to 50 µL PBS in the first column in a 96-well v-shaped bottom plate (Molecular Devices, Sunnyvale, CA, USA) and the solution was incubated for 30 min at 37 °C. A serial of dilutions were prepared as 1:2 and 50 µL SRBC (2%) suspension was added to each well. The plates were read on a microplate reader at 650 nm to evaluate the total immunoglobin (Ig) titer, 30 min after incubation at 37 °C. Immediately, the well with a distinct SRBC bottom was considered as the end-point of the titer for agglutination. In addition to evaluate IgG titer, 50 µL 2-mercaptoethanol (0.01 M) in PBS was used and then followed the previous procedure. The difference between total Ig and IgG titers was considered as IgM titer. Anti-SRBC titers were measured and expressed as log_2_ of the reciprocal of the last dilution after complete agglutination (Pilevar et al., 2011 ▶).

**Tissue sampling and section preparation**

At the end of study, 2 birds from each treatment were sacrificed for histology of jejunum. The tissue samples (2 cm) were taken from the midpoint between the point of entry of the bile duct and Meckel's diverticulum in jejunum (Awad et al., 2005a ▶). 

The segments were flushed by 0.9% saline and fixed in 10% formaldehyde buffer solution for 48 hr. The tissue processing consisted of serial dehydration (soaked samples were rinsed 3 times in absolute alcohol), imbedding, and fixing in paraffin. Tissue sections were harvested from 5 mm pieces with 5 μm thickness (fine three cross-sections from each sample) by microtome, fixed on slides and stained with Gill’s hematoxylin and eosin (Arshami and Ruttle, 1988 ▶). Eight sections per sample were utilized for a histopathological study using an image analyzer (Nikon Cosmozone 1S, Nikon, Tokyo, Japan).

**Measurement of villus parameters and intestinal layer thicknesses**

The images were analyzed using the stereological image software, Cast Image System (Version 2.3.1.3). The villus length (VL) was measured from the villus tip to the bottom, not including the intestinal crypt. A total of 16 villi per section were measured in each replicate and four VL were averaged as the mean of villus length. Similarly, the crypt depth (CD) from the crypt-villus junction to the base of the crypt, VL/CD and villus width (VW) at mid-villus height were measured. The villus surface (VS) and the villus surface area (VSA) were calculated using the villus number (VN) in 100 μm^2^ and the following formulas (Sakamoto et al., 2000 ▶): 

VS = (2π) × (VW/2) × (VL) and VSA = VN (in 100 μm^2^) × VS

The villus is assummed a cylindrical shape that its surface area (a rectangular) can be calculated by multiplying length of villus (VL) and circumference of villus base (2πR). The R is radius of villus base and equal to half of villus width (VW/2). The thickness of the lamina propria (LPT) and the muscle layer thickness (MLT) in the jejunum wall were measured accordingly.

**Statistical analysis**

The data obtained is shown in mean±SD. Statistical analysis was performed using one-way analysis of variance (ANOVA) and for post-hoc comparisons, Duncan’s multiple range test was applyed. The significant level was reported at 5%.

## Results

**Blood parameters**

The results of blood parameters are presented in Table 1. The levels of triglycerides, cholesterol, and LDL decreased by increasing curcumin doses (p<0.05). However, the levels of glucose, total protein, and albumin decreased slightly as the curcumin level increased. The curcumin failed to affect on creatin kinase, calcium, and phosphorous during different weeks.

**Anti-SRBC titers**

The results of immune responses during the 1^st^ and 2^nd^ injections are shown in Table 2. The overall results showed no significant differences between treatments but the anti-SRBC titers were higher in the 2^nd^ inoculation compared with the 1^st^ one. The total anti-SRBC titers were highest at the 0.5% and 1.5% curcumin in the 2^nd^ inoculation. The IgG titers were highest at the 1.5% curcumin in the 1^st^ and 2^nd^ inoculations. The IgM titers increased in all treatments at the 1^st^ inoculation but in the 2^nd^ inoculation, the 0.5% and 1.5% curcumin increased IgM with the highest for 0.5%.

**Table 1:** Effects of different levels of curcumin powder (CP) on blood parameters in hens

**Treatments** 1	** 1**	**2**	** 3**	** 4**	**SEM**	**p-value**
**Triglyceride mg/dL**	97^a^	83.5^ab^	81.7^b^	79.9^b^	3.47	0.017
**Cholesterol mg/dL**	126.55^a^	108.12^ab^	103.36^ab^	96.01^b^	5.84	0.018
**LDH U/L**	243^a^	219.7^ab^	189.7^ab^	173.7^b^	15.95	0.041
**Glucose mg/dL**	201.37	188.03	185.02	170.84	13.14	0.465
**Protein Total g/L**	2.82	2.35	2.02	2.72	0.25	0.130
**Albumin g/L**	3.5	3.00	2.87	3.02	0.32	0.571
**Creatine Kinase U/L**	417.15	378.0	391.5	428.28	41.03	0.813
**Calcium mg/dL**	10.4	10.67	10.00	10.49	0.72	0.926
**Phosphorous mg/dL**	6.78	6.89	6.66	7.01	0.05	0.964

1 Treatments were included: 1=0.0% CP, 2=0.5% CP, 3=1.5% CP, and 4=2.5% CP or 5, 15, and 25 g curcumin powder/kg feed, respectively.

ab Means with different superscripts in a row are significantly different (p<0.05).

**Villus parameters and intestinal layers**

The results of villus parameters and intestinal layer thicknesses are presented in Table 3. 

The overall inclusion showed no significant improvement in villus parameters except slight increase in the villus length and villus surface area at 1.5% curcumin. The 1.5% curcumin increased the muscle layer thickness (p<0.05), whereas the 0.5% and 2.5% curcumin showed slight increase compared with the control group.

**Table 2:** Effects of different levels of curcumin powder (CP) on antibody titres at 7 days after 1^st^ and 2^nd^ SRBC injections in hens

	**Seven days after 1** ^st^ ** injection**			**Seven days after 2** ^nd^ ** injection**		
**Treatments** 1	**Total anti-SRBC**	**IgG**	**IgM**	**Total anti-SRBC**	**IgG**	**IgM**
**1 **	9.1	7.3	1.8	9.8	7.8	2.0
**2 **	9.0	6.8	2.2	10.1	6.7	3.4
**3 **	9.7	7.7	2.0	10.7	8.5	2.2
**4 **	9.0	6.9	2.1	9.0	7.3	1.7
**SEM**	0.58	0.55	0.45	0.65	0.73	0.5
**p-value**	0.799	0.655	0.935	0.353	0.388	0.135

1 Treatments were included: 1=0.0% CP, 2=0.5% CP, 3=1.5% CP, and 4=2.5% CP or 5, 15, and 25 g curcumin powder/kg feed, respectively.

**Table 3:** Effects of different levels of curcumin powder (CP) on villus and jejunum layer parameters in hens

**Treatments** 1	**Villus length (µm)**	**Villus width (µm)**	**Villus surface area (µm** ^2^ **)**	**Crypt depth (µm)**	**Lamina properia (µm)**	**Muscularis thickness (µm)**
**1**	848.89	149.44	394565	164.42	45.29	227.18^b^
**2**	835.89	125.14	331738	185.15	41.53	236.1^ab^
**3**	922.09	136.34	396246	165.96	42.74	322.82^a^
**4**	716.91	148.71	382019	151.65	45.61	257.35^ab^
**SEM**	114.57	17.00	65090	14.87	2.96	21.85
**p-value**	0.657	0.715	0.883	0.487	0.724	0.036

1 Treatments were included: 1=0.0% CP, 2=0.5% CP, 3=1.5% CP, and 4=2.5% CP or 5, 15, and 25 g curcumin powder/kg feed, respectively.

ab Means with different superscripts in a column are significantly different (p<0.05).

## Discussion

Curcumin powder is a yellowish spice known to possess antimicrobial and antioxidative properties. In this study, statistical analysis of data showed different effects of curcumin on blood parameters, anti-SRBC titer, and histomorphology of jejunum in hens. Animal studies documented the antioxidative and hypolipidemic effects of curcumin on lipid metabolism, especially anti-hypercholesterolemia.

Our results showed reduction of cholesterol, triglycerides, and LDL, having negative trend with treatments (p<0.05). Similarly, the graded curcumin powder (0.05 to 0.20%) significantly decreased serum triglyceride, total cholesterol, and LDL but increased HDL in laying hens at 104 weeks of age (Kermanshahi and Riasi, 2006 ▶). Moreover, it has been reported that hens (28 wk) fed curcumin at 1.0% significantly decreased yolk total lipid by 13.95% (Radwan et al., 2008 ▶). However, recent study showed a significant reduction of triglycerides with no effects on other lipid parameters in subjects treated with curcuminoids (1 g/day) (Mohammadi et al., 2012 ▶). This discrepancy with our study and others may explain the variation among the experimental units or the source of curcumin. The significant reduction of serum lipids are clearly indicated the hypolipidemic effects of curcumin which is dose-dependent. 

In our study, we observed slight reduction of total protein, albumin, and glucose with increasing curcumin dose. However, it has been reported that curcumin exhibits hypoglycemic effects in type 2 diabetic mice and diabetic albino rats, (Nishiyama et al., 2005 ▶; Arun and Nalini, 2002 ▶). Moreover, another study showed that curcumin can modulate the glucose level in diebetic subjects (Srinivasan, 1972 ▶). Thus, accoding to our study and other reports, curumin can reduce and regulate blood sugar level in healthy and diebetic subjects. 

In this study, we assessed the immunomodulatory properties of curcumin in hens during weeks 58-65 of age (Table 2). We found no significant differences between treatments, but the anti-SRBC titers were higher in the 2^nd^ inoculation compared with the 1^st^ one with the highest for 0.5% and 1.5% curcumin. This finding indicated that curcumin slowly built up the immune system to respond to inoculation after the 2^nd ^injection. However, it has been reported that different levels of curcumin powder (0.25 to 0.75%) significantly increased IgA, IgM, and IgG titers but decreased the ratio of monocytes in broilers at 42 days of age (Emadi and Kermanshahi, 2007 ▶). In our study, the IgG titer was the highest at 1.5% curcumin and the IgM titers increased in all treatments at the 1^st^ and 2^nd^ inoculations with the highest for 1.5%. Similarly, another study found that dietary curcumin at 40 mg/kg enhanced IgG titer in rats (). Moreover, it has been reported that feeding 1 g of curcumin/kg to mice increased mucosal CD4+T cells and B cells in the intestine, indicating that curcumin can modulate lymphocyte mediated immune functions (). However, adding curcumin (90 or 135 ppm and 180 ppm) to the broiler diets reduced H:L at 28 and 42 days of age respectively (p<0.05) with no significant effect on NDV titer (Kijparkorn and Angkanaporn, 2003 ▶). In our study, some improvements were observed in humoral immunity, which was dose-dependent.

In general, improving digestion and absorption depends on the stimulation of appetite, feed intake, improvement of endogenous digestive enzyme secretion, and activation of intestinal layers and villi. Adding curcumin to diet may provide sufficient improvement through morphological changes of intestinal layers and villi in jejunum to maintain growth and development. It is suggested that the stabilizing effect on intestinal morphology may be associated with intermediate nutrient metabolism (Jamroz et al., 2003 ▶). The overall inclusion of histology in this study showed various effects by treatments (Table 3). The 1.5% curcumin increased the thickness of muscle layers (p<0.05). The 1.5% treatment also increased villus length and villus surface area as well. The lamina properia thickness and crypt depth increased in 2.5% and 0.5% curcumin, respectively. The digestive activity of curcumin is perhaps mediated via improvement of layers and villus characteristics in jejunum. 
